# Critical temperature requirement for the germination and establishment of mungbean (*Vigna radiata* L.) in temperate environments

**DOI:** 10.3389/fpls.2026.1693408

**Published:** 2026-02-06

**Authors:** Sachesh Silwal, Audrey J. Delahunty, Ashley J. Wallace, Sally Norton, Alexis Pang, James G. Nuttall

**Affiliations:** 1 Agriculture Victoria, Department of Energy, Environment and Climate Action (DEECA), Horsham, VIC, Australia; 2 School of Agriculture, Food and Ecosystem Sciences (SAFES), Faculty of Science, The University of Melbourne, Parkville, VIC, Australia; 3 Agriculture Victoria, Department of Energy, Environment and Climate Action (DEECA), Irymple, VIC, Australia; 4 Australian Grains Genebank, Agriculture Victoria, Department of Energy, Environment and Climate Action (DEECA), Horsham, VIC, Australia; 5 School of Applied Systems Biology, La Trobe University, Bundoora, VIC, Australia; 6 Birchip Cropping Group, Birchip, VIC, Australia

**Keywords:** cold tolerance, constant temperature, diurnal temperature, germination, germination rate, low soil temperature, temperate

## Abstract

Mungbean (*Vigna radiata* (L.) R.Wilczek.) is an important annual legume cultivated in subtropical regions for its high-protein grains. However, it is susceptible to low temperatures (<20°C) during germination and establishment, which results in substantial yield loss. Early growth stages are crucial for successful cultivation in cooler climates to enable an optimal sowing window and effective establishment. This study aimed to identify cold-tolerant mungbean genotypes adapted to low-temperature germination (<20°C), particularly in southern Australia during November-December. The effects of temperature (14, 17 and 20°C) and soil water availability (40 and 80% of field capacity) on the germination and emergence of mungbean genotype were investigated through three experiments. In Experiment 1, thirty-two genotypes were evaluated for germination at constant temperatures of 14, 17 and 20°C using germination paper towels in a controlled environment. Additionally, in Experiment 2 and 3 in controlled environment experiments using soil-filled pots were conducted to determine the effect of temperature under constant (14, 17, and 20°C) and a range of diurnal temperature regimes (10–18°C, 13–21°C, and 16–24°C), ensuring that the average temperature for each treatment remained at 14, 17, and 20°C respectively. These temperatures were tested in factorial combination with soil water status (40 and 80% of field capacity) on the germination and emergence of commercial varieties Jade-AU and Celera II-AU. Germination occurred at all tested temperatures, with the highest germination percentage observed at 20°C on paper towels. Genotypes Putland, Jade-AU, Bari Mung-3, Bari Mung-4, Satin II, and Bina Mung-8 showed no significant differences in germination rates among the 14, 17, and 20°C temperature treatments, with average germination percentages exceeding 80% in the paper-towel evaluation. The median germination rate observed was highly variable (2–16 days) across genotypes in response to temperature treatment. The estimated base temperature of Celera II-AU and Jade-AU was 8.6 and 9.8°C, respectively. Seedling emergence was faster and higher for Celera II-AU than Jade-AU across the diurnal and constant temperatures. The germination was observed at both diurnal and constant temperature treatments of 20, 17, and 14°C. However, no emergence was observed at a constant temperature of 14°C for varieties Jade-AU and Celera II-AU. These findings suggest that mungbean can be successfully sown in early spring of southern Australia if soil temperature is at least 17°C. This research provides valuable insights for future breeding programs, germination studies, and sowing date recommendations in temperate environments.

## 1 Introduction

Mungbean (*Vigna radiata* (L.) R.Wilczek.) is an annual grain legume that is an integral part of major cropping systems, particularly in Asia. Mungbean is rich in protein, vitamins, and minerals, and can be eaten as a whole seed, sprouted, or processed into various forms such as noodles, porridge, or soups. They have a short growing period (60–90 days under subtropical conditions) (Sengupta, 2018), can be beneficially used as a cover crop or intercrop between cereal crops, and can be grown in places with poor soil fertility and limited water availability. Incorporating mungbean into the cropping system also helps to increase soil nitrogen by atmosphere fixation (30–251 kg/ha) (Islam et al., 2021).

Within Australia, mungbean is normally produced in northern sub-tropical regions, where the rainfall is summer-dominant, with 90% exported to Asia and America (Australian Mungbean Association, 2021; AEGIC, 2024). In addition to increasing production within current regions through optimising agronomic practices and adaptability (breeding), there is the potential to expand into new regions. One potential growing region is southern Australia (e.g., Victoria), most of which is characterised as a temperate climate with winter-dominant rainfall (Bureau of Meteorology, 2023). In southern Australia, the cropping system is predominantly winter crops, such as wheat, barley, lentil, and canola, and inclusion of mungbean as a summer crop may be possible. However, a significant constraint in expanding crop cultivation into new growing environments is the compatibility of the genotype with varying climatic and temperature profiles. One notable challenge associated with mungbean cultivation in southern Australia is the lower soil temperature encountered in spring, which is the sowing window (Delahunty et al., 2020).

Temperatures in southern Australia are cooler than in the subtropical regions of Queensland and northern New South Wales. An analysis of soil temperatures in Victoria determined that the temperature is borderline low for mungbean germination during spring (Silwal et al., 2025). The average soil temperature during October–November stays below 20 °C across Victoria. For instance, at the northern site of Ouyen (35°00′32′′ S, 142°15′00′′ E) and the southern site of Hamilton (37°49′38′′ S, 142°04′12′′ E), the soil temperature remains below 20°C until the end of November (Delahunty et al., 2020). Previous research (Szczerba et al., 2021) has shown that low temperatures inhibit soybean germination; for the cultivars tested, no germination occurred at 10°C, while moderate germination (17–53%) was observed at 15°C, and high germination rates (97–100%) were achieved at 25°C, 48 hours after sowing.

The successful germination and early establishment of mungbean seeds are crucial for vigorous plant growth, high yield potential, and overall crop productivity. Establishing 30 plants/m^2^ is optimum for higher yields in mungbean cultivation in subtropical environments (Rachaputi et al., 2015). Low soil water and temperature result in a reduced germination rate and final germination percentage. Mungbean germination relies on exceeding the base temperature (10°C) for 3–4 consecutive days, with higher temperature increasing the emergence rate (Fyfield and Gregory, 1989). Furthermore, the lowest base temperature for mungbean growth reported is 7.5°C, and soil temperature exceeding 15°C is required for effective germination (Chauhan and Williams, 2018). Optimal temperature plays a crucial role in mungbean germination. In a controlled experiment, when water was not a limiting factor, it was determined that the optimal temperature range for mungbean germination is between 30 and 40°C, with the fastest rate observed at 40°C (Fyfield and Gregory, 1989). Conversely, research under laboratory conditions found that the time required to germinate 50% of seeds increased significantly below 14°C (Simon et al., 1976), with the lowest temperature at which 50% germination was achieved being 11.5°C. Another important factor for germination is water, with observations indicating at least 50% seedling emergence when the soil water potential was greater than −0.5 MPa (Fyfield and Gregory, 1989), which is equivalent to 50–70% plant available water for clay and 10–20% plant available water for sand. This sensitivity to soil water status becomes more pronounced under low-temperature (<20°C) and drought conditions. In cooler conditions, water uptake by seeds slows considerably, leading to slower metabolic processes and a more extended imbibition period to initiate the germination process (Szczerba et al., 2021). If soil water decreases below the −0.5 MPa threshold during this time, germination may be delayed or fail. Drought conditions further reduce soil water potential, particularly affecting sandy soils with low water-holding capacity (Yu et al., 2023). While clay soil near field capacity can buffer periods with no rainfall, water also becomes limited during extended dry spells. Thus, the combination of low temperatures and drought significantly impacts germination, highlighting the critical need for consistent water availability for germination, emergence and early seedling establishment.

The variation among mungbean genotypes in their response to cool soil temperatures in their response to cool soil temperatures during spring sowing is therefore critical for achieving reliable germination, particularly under low temperature (<20°C) sowing conditions. A crop is sown when soil temperatures are favourable, as the timing of sowing plays a vital role in ensuring rapid and uniform establishment. This highlights the importance of identifying and understanding the effects of lower temperatures on mungbean seedling emergence and the ability to support sowing opportunity during October–December in southern Australia.

In this study, mungbean response to low temperature on germination and emergence was evaluated to test the following hypotheses: i) low temperature ranges (<20°C) typically encountered in south-eastern Australia during October–December will inhibit germination and establishment of mungbean, and ii) genetic diversity can enable improved adaptation to cooler soil conditions. The knowledge generated by this study will help to identify potential genetic diversity for improving cold tolerance of mungbean, and improved adaptation to temperate environments.

## 2 Materials and methods

The experiments were conducted at the Agriculture Victoria Smart Farm, Victoria, Australia (36°43’14.2”S, 142°10’24.4”E) under controlled environment conditions using growth cabinets (STERIDIUM, pledt-rh-2900). The seed tested was sourced from the Australian Grains Genebank (AGG), except for the reference genotypes Celera II-AU and Jade-AU, which were commercial, certified seed obtained from the Australian Mungbean Association. Mungbean genotypes Jade-AU and Celera II-AU (commercial cultivars) were selected as reference genotypes in Experiment 1 and were evaluated for soil-based germination in Experiment 2 and 3 due to their wider commercial cultivation in Australia. These reference genotypes have contrasting plant architecture, growth habit and grain type (market class). Jade-AU is large seeded (74 mg) while Celera II-AU is small seeded (41 mg) (**Table 1**).

**Table 1:** Details of mungbean genotypes tested for germination at 14, 17 and 20°C (Experiment 1).

Genotype name	Seed weight (mg) (mean ± sd)	Country of origin	AGG accession identifier	Accession DOI
BARI MUNG-2	44 ± 7	Bangladesh	AGG 325773 MUNG	https://doi.org/10.18730/1N4ZEZ
BARI MUNG-3	43 ± 6	Bangladesh	AGG 325774 MUNG	https://doi.org/10.18730/1N4ZF*
BARI MUNG-4	41 ± 6	Bangladesh	AGG 325775 MUNG	https://doi.org/10.18730/1N4ZG~
BARI MUNG-5	53 ± 9	Bangladesh	AGG 325776 MUNG	https://doi.org/10.18730/1N4ZH$
BARI MUNG-6	65 ± 11	Bangladesh	AGG 325777 MUNG	https://doi.org/10.18730/1N4ZJ=
BINA MUNG-5	58 ± 11	Bangladesh	AGG 325778 MUNG	https://doi.org/10.18730/1KY6W$
BINA MUNG-6	52 ± 8	Bangladesh	AGG 325779 MUNG	https://doi.org/10.18730/1KY6X=
BINA MUNG-7	66 ± 11	Bangladesh	AGG 325780 MUNG	https://doi.org/10.18730/1KY6YU
BINA MUNG-8	51 ± 8	Bangladesh	AGG 325781 MUNG	https://doi.org/10.18730/1KY6Z0
BU MUNG-4	50 ± 9	Bangladesh	AGG 325782 MUNG	https://doi.org/10.18730/1KY701
CELERA	46 ± 7	Australia	AGG 307933 MUNG	https://doi.org/10.18730/18DZ2K
CELERA II-AU	48 ± 8	Australia	AGG 325316 MUNG	https://doi.org/10.18730/18RK9W
Celera II-AU (Check)	41 ± 6	Australia	Not Applicable	Not Applicable
CRYSTAL	90 ± 14	Australia	AGG 325314 MUNG	https://doi.org/10.18730/18RK7T
DELTA	84 ± 13	Australia	AGG 321623 MUNG	https://doi.org/10.18730/1KY6V~
EMERALD	79 ± 13	Australia	AGG 307985 MUNG	https://doi.org/10.18730/18E0N~
GREEN DIAMOND	49 ± 8	Australia	AGG 308033 MUNG	https://doi.org/10.18730/18E257
JADE-AU	86 ± 13	Australia	AGG 325315 MUNG	https://doi.org/10.18730/18RK8V
Jade-AU (Check)	74 ± 12	Australia	Not Applicable	Not Applicable
PUTLAND	51 ± 6	Australia	AGG 307979 MUNG	https://doi.org/10.18730/18E0FV
SATIN II	75 ± 17	Australia	AGG 325317 MUNG	https://doi.org/10.18730/18RKAX
VI064685	43 ± 6	India	AGG 327273 MUNG	https://doi.org/10.18730/1E6A3E
VI064686	39 ± 6	India	AGG 327274 MUNG	https://doi.org/10.18730/1E6A5G
VI064688	43 ± 8	India	AGG 327276 MUNG	https://doi.org/10.18730/1E6A8K
VI064689	35 ± 4	India	AGG 327277 MUNG	https://doi.org/10.18730/1E6AAN
VI064691	56 ± 10	India	AGG 327279 MUNG	https://doi.org/10.18730/1E6ADR
VI064692	45 ± 8	India	AGG 327280 MUNG	https://doi.org/10.18730/1E6AFT
YEZIN-1	63 ± 16	Myanmar	AGG 325783 MUNG	https://doi.org/10.18730/1KY712
YEZIN-11	68 ± 11	Myanmar	AGG 325785 MUNG	NA
YEZIN-14	70 ± 11	Myanmar	AGG 325786 MUNG	https://doi.org/10.18730/1KY734
YEZIN-6	40 ± 8	Myanmar	AGG 325787 MUNG	https://doi.org/10.18730/1KY745
YEZIN-9	77 ± 13	Myanmar	AGG 325784 MUNG	https://doi.org/10.18730/1KY723

DOI, Digital Object Identifier; NA, Not available.

The effects of temperature (14, 17 and 20°C) and soil water availability (40 and 80% of field capacity) on the germination and emergence of mungbean genotype were investigated through three experiments: Experiment 1 (assessment of seed germination with a paper towel across constant temperature treatments); Experiment 2 (soil-based seed germination and emergence across constant temperatures and soil water status); and Experiment 3 (soil-based seed germination and emergence across diurnal temperatures and soil water status).

### 2.1 Experiment 1: assessment of seed germination with paper towel across constant temperature treatments

The experiment was conducted on the AGG core collection of 32 genotypes of mungbean from Bangladesh, Myanmar, Australia, and India. All the genotypes were multiplied in the field during summer 2020 at Horsham (36°44’57” S, 142°06’59” E and 128 m.a.s.l.), except for the reference genotypes Jade-AU and Celera II-AU (sourced from commercial certified seed supplier). Additionally genotypes VI064685, VI064686, VI064688, VI064689, VI064691 and VI064692 were sourced directly from AGG which was multiplied in Queensland during summer of 2021 (**Table 1**).

**Figure 1:**
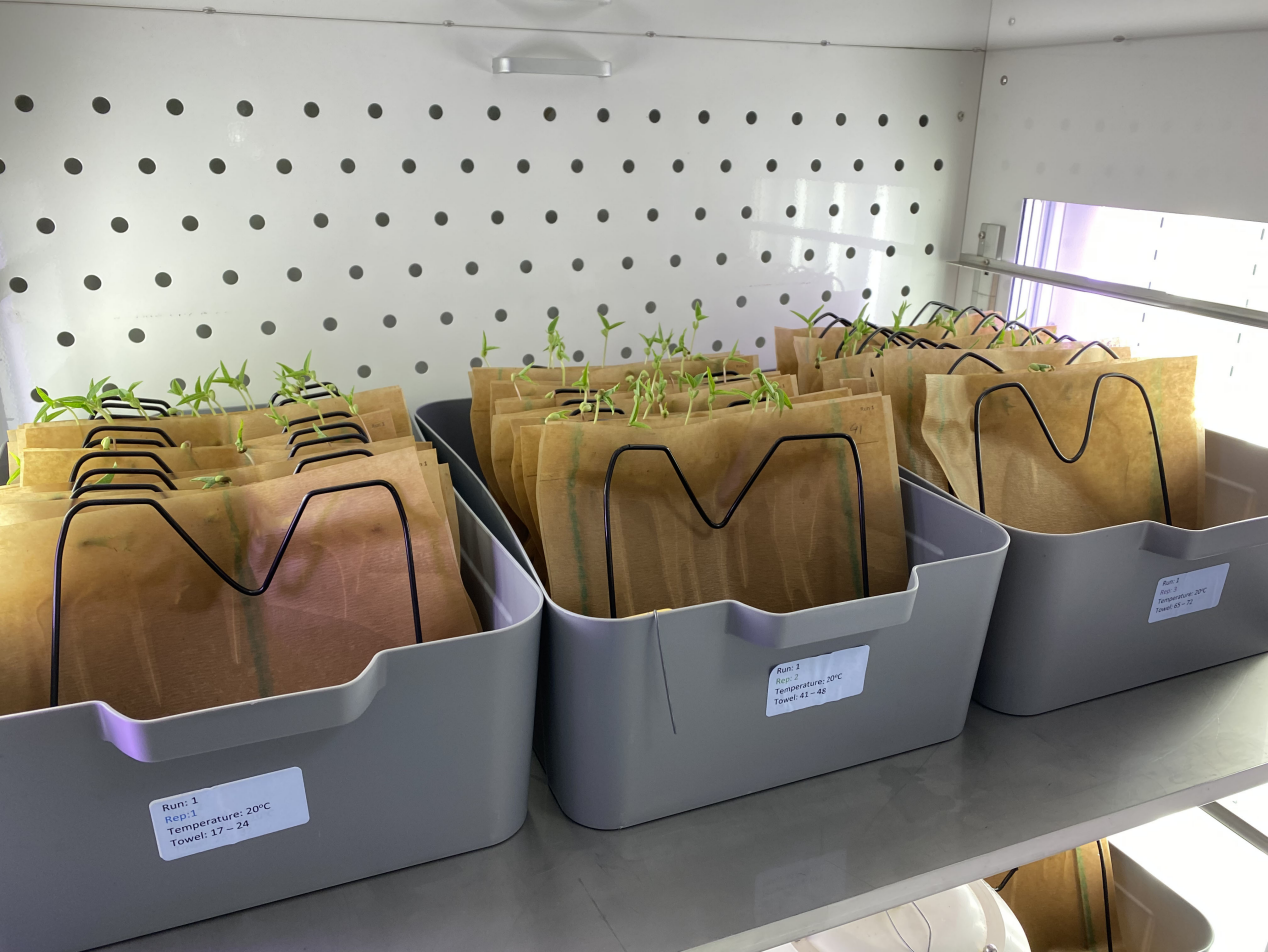
Germination stand and paper setup within growth cabinet at Horsham, Victoria (Experiment 1).

The genotypes were evaluated at constant temperature treatments of 14, 17, and 20°C with a 10-h photoperiod (8 h day and 2 h twilight) in climate-controlled growth chambers (STERIDIUM, PLEDT-RH-2900). The observed temperatures (mean ± sd) of constant temperature treatments for 14, 17 and 20°C were 14 ± 0.2, 17 ± 0.1 and 20 ± 0.1°C respectively; and observed relative (mean ± sd) humidity were 65 ± 4.5, 60 ± 3.1, and 55 ± 1.4% respectively. The 8 h day is with 100% light intensity and the twilight period of 2 h (1 h before and 1 h after 8 h day) with light intensity of 50%. The study was performed from May 2021 to August 2023. Seeds were germinated using Anchor Paper seed germination papers (No. 38) in stands measuring 24 cm in length, 20 cm in width, and 20 cm in height, which were used to hold the germination towels (**Figure 1**). The germination stands were placed in plastic trays 42 cm in length, 28 cm in width, and 13 cm in height. Each tray was filled with 3 L of reverse osmosis (RO) water to soak germination towels, ensuring at least 3 cm of the towels were submerged in the water. The RO water was added to maintain the water level during the experiment.

The experiment was conducted using a randomised complete block design within controlled environment growth cabinets, with five independent experimental runs for each temperature treatment. These runs served as independent temporal repetitions to ensure the reproducibility of the results. In each run, a subset of eight genotypes (six test genotypes and two reference genotypes) was evaluated. To validate the consistency across these repetitions, ‘run’ and its interactions were included as random effects in the REML model, and their significance was assessed using Likelihood Ratio Tests (LRT). Each run comprised six test genotypes and two reference genotypes (Celera II-AU (check) and Jade-AU (check)), with each genotype replicated four times within each run, i.e. four biological replicates per genotype within each run. To facilitate comparison of genotypes across runs and to account for temporal variation, two reference genotypes, Celera II-AU (check) and Jade-AU (check), were included in each run (*n* = 20, biological replicates per temperature). The remaining test genotypes were evaluated in specific runs (*n* = 4 biological replicates per genotype per temperature). The independence of these five runs ensured that the evaluation was not limited to a single point in time, fulfilling the requirement for independent experimental repetitions.

Each tray was treated as a block or replication within each run of a temperature treatment. Eight genotypes were randomised and evaluated in each tray. Ten seeds from each genotype were placed in a germination towel at a depth of 3 cm and positioned within the germination stand. The seeds were selected at random, and each seed was weighed before being placed on the growth germination towel.

Seed germination was defined as the growth of the radicle with at least 3 mm (Lamichhane et al., 2020). In this stage, the seeds absorb water from the surrounding and start biochemical events crucial for the growth of plumule and radicle. Germination percentages were determined at 3, 5, 7, 10, 14, 17, and 21 days after sowing (DAS), with the lengths of the radicle and hypocotyl measured using a ruler. The unit of measurement was millimetres, and the level of precision was 0.1 cm. The total length of the radicle and hypocotyl was expressed as root length.

#### 2.1.1 Germination percentage

Germination percentage refers to the proportion of seeds tested that sprout into seedlings and is expressed by the formula:


$$Germination\;percentage\;(\%)=\frac{Number\;of\;seeds\;germinated\times 100}{Total\;number\;of\;seeds}$$

#### 2.1.2 Germination rate

Germination rate (GT_50_), or the median germination time, is the time to reach 50% of maximum germination in elapsed days. The germination rate (GT50) was calculated based on Coolbear et al. (1984):


$$GT_{50}=t_{i}+[\frac{(N+1)/2-n_{i}}{n_{j}-n_{i}}].(t_{j}-t_{i})$$

where N is the final number of seeds germinated and n_i_, n_j_ the total number of seeds germinated by adjacent counts at time t_i_, t_j_, where n_i_ < (N + 1)/2 < n_j_.

#### 2.1.3 Base temperature

A regression plot of reciprocal GT_50_ (1/GT_50_) against temperature was plotted for a linear relationship for temperature. The extrapolation of linear regression back to temperature axis (x-intercept) gave estimates of base temperature (T_b_) or the theoretical minimum temperature for germination.

#### 2.1.4 Percentage of seed germination after sowing

The data was analysed using generalised linear mixed model (GLMM) to estimate percentage of seed germination at each DAS in GenStat 22^nd^ edition (Schall, 1991; VSN International, 2022) at each DAS. The fixed effects of seed weight, temperature, genotype, and the interaction between temperature and genotype were tested. Run, temperature, replication, and germination towel were included as random effects. A binomial distribution with a logit link was used.

### 2.2 Experiment 2: soil-based seed germination and emergence across constant temperatures and soil water status

A factorial germination experiment was conducted under controlled growth cabinets over 21 days to test the effect of two mungbean genotypes (Jade-AU and Celera II-AU), two soil water statuses (40 and 80% field capacity) and three constant temperature treatments (14, 17 and 20°C). The experiment followed a randomised complete block design with four biological replicates (*n* = 4) of each factorial combination (independent randomisation of 16 experimental units i.e. 2 genotypes x 2 soil water statuses x 4 replications) within each temperature treatment in each cabinet ensured the independence of the biological responses and provided the degrees of freedom necessary for robust statistical inference. The observed temperatures (mean ± sd) of constant temperature treatments for 14, 17 and 20°C were 14 ± 0.2, 17 ± 0.3 and 20 ± 0.2°C, respectively; and observed relative (mean ± sd) humidity were 57 ± 3.8, 54 ± 2.2, and 58 ± 2.1% for 14, 17 and 20°C, respectively.

The experiment was conducted in controlled growth cabinets with a 10 h photoperiod (8 h day and 2 h twilight). The pots were filled with a topsoil (0–15 cm) of Vertosol soil (Isbell and the National Committee on Soil and Terrain, 2021) collected from Horsham, Victoria (36°44’57” S, 142°06’59” E), Australia. The soil was air dried (40°C for 4 days) and passed through a 5 mm sieve. Soil was packed in large sealed square pots (160 mm width and 240 mm height) with 5500 g of soil per pot, which equated to a bulk density of 1.49 g/cm^3^. The field capacity of the soil was 46% (i.e., 0.68 g/cm^3^). Pots were pre-watered to a weight equivalent of 35 and 70% of field capacity and brought to 40 and 80% of field capacity after seed sowing. Pots were watered using metered amounts of reverse osmosis (RO) water every three days to maintain the water content of pots at 40 and 80% of field capacity throughout the experiment. Pots were left for three days in the growth chambers to allow acclimatisation of soil and water to set temperatures before planting. Additionally, the water was stored inside the growth cabinet at least 24 h before adding to maintain the set temperature. Five mungbean seeds were planted per pot, and each pot received a basal application of Granulock Z fertiliser (Incitec Pivot Fertilisers, Australia) @ 0.22 g/pot.

Seedling emergence and shoot length were counted daily for up to 21 days after sowing. Emergence of seedling refers to the emergence of hypocotyl above the ground level. In case of mungbean, emergence occurs when the hypocotyl elongates rapidly, arches upward pushing cotyledons out of the ground (Epigeal). Emergence was recorded when the cotyledons were seen on the soil surface. Shoot length was measured as the height of seedlings above the ground. Root length and final germination were assessed on 21 DAS by gently washing the soil off.

### 2.3 Experiment 3: soil-based seed germination and emergence across diurnal temperatures and soil water status

A factorial germination experiment was conducted to test the effect of two genotypes of mungbean Jade-AU and Celera II-AU, under two different soil water statuses (40 and 80% field capacity) and three diurnal temperature regimes (10 to 18, 13 to 21, and 16 to 24°C), ensuring that the average temperature for each treatment remained at 14, 17, and 20°C respectively throughout the 21-day experiment (**Supplementary Figures 1**, **2**). The experiment employed a randomised complete block design with four biological replicates of each factorial combination and was repeated twice as independent experimental runs. This resulted in a total of eight biological replicates (*n* = 8) per treatment combination across the two repetitions. The observed mean temperatures (mean ± sd) for diurnal temperatures were 14 ± 3.6, 17 ± 3.6 and 20 ± 3.6°C; and observed relative (mean ± sd) humidity were 57 ± 3.7, 53 ± 2.2, and 58 ± 2.1% for 14, 17 and 20°C, respectively. The maximum temperature was at 100% light intensity (day) for 8 h, and the minimum temperature was at 0% light intensity for 8 h (night). The temperature changed during cycle with linear transitions of 4 h in between minimum and maximum temperature with light intensity of 50% (twilight period).

The experimental setup was equivalent to that of Experiment 2 including soil type, watering regime, fertilizer rate, except for the temperature treatment and photoperiod. The parameters recorded were consistent with those in Experiment 2.

### 2.4 Data analysis

For Experiment 1, the residual maximum likelihood (REML) method was used to analyse the responses of genotype and temperature effects on various parameters. Temperature and genotype were treated as fixed effects, while run, temperature, replication, and germination towel were included as random effects. The effect of independent experimental repetitions (runs) was assessed using Likelihood Ratio Tests (LRT), and the run × temperature interaction was accounted for in the random effects to pool the data across the independent runs. All the analyses were performed using Asreml-R package version 4.2.0.355 (The VSNi Team, 2023) in R 4.4.2 and GenStat 22^nd^ edition (VSN International, 2022). The significance of fixed effects was assessed using the Wald test at a 0.05 significance level.

For Experiments 2 and 3, the effects of temperature, genotypes, and soil water status were analysed using a three-factor analysis of variance (ANOVA), with days after sowing (DAS) treated as a repeated measures factor using GenStat 22^nd^ edition (VSN International, 2022). These data were presented as descriptive and summary statistics. Means were compared using Fisher’s least significant difference (LSD) test at a 0.05 significance level.

## 3 Results

### 3.1 Experiment 1: assessment of seed germination with paper towel across constant temperature treatments

#### 3.1.1 Germination percentage

**Figure 2:**
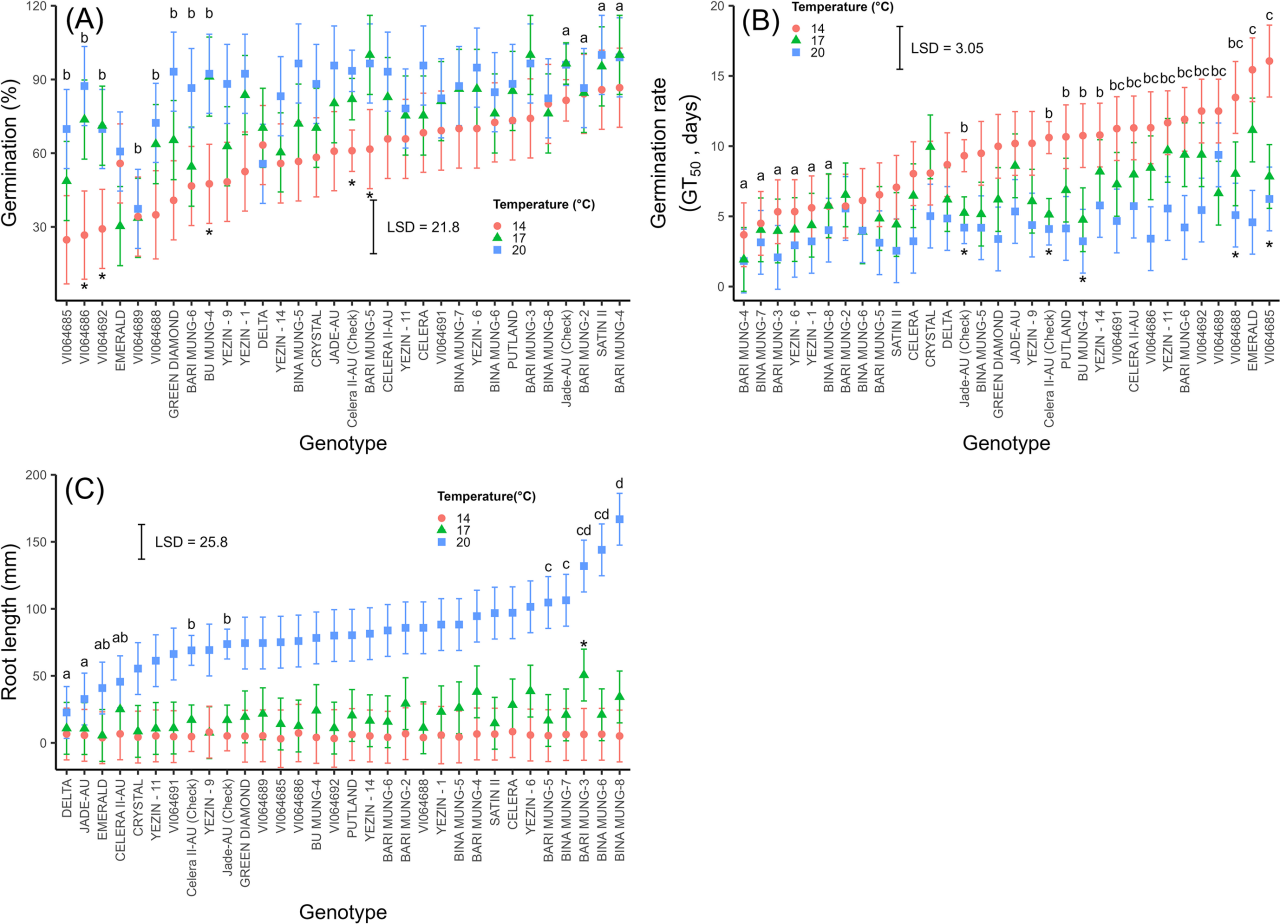
The interaction effect of temperature (14, 17 and 20°C) and mungbean genotypes at 21 DAS on **(A)** germination percentage, **(B)** germination rate (GT_50_), and **(C)** root length under controlled growth conditions (Experiment 1). During experiment 1, the average temperatures were 14 ± 0.2, 17 ± 0.1 and 20 ± 0.1°C for the constant temperature treatments of 14, 17 and 20°C, respectively. Points represent REML (Asreml-R) estimated means and error bars represent the 95% confidence interval for each genotype at each temperature treatment. The confidence interval between genotypes differs because the reference genotypes were evaluated across all five independent experimental runs (*n* = 20), whereas individual test genotypes were evaluated within a single run (*n* = 4). A Likelihood Ratio Test (LRT) confirmed no significant main effect of run (*p* > 0.05), allowing for the valid estimation of BLUEs across the integrated experimental structure. Genotypes with highest germination percentage **(A)** are denoted by letter “a” and genotypes with different letters are significantly different from “a” at 14°C, according to Wald test at a *p ≤* 0.05. Genotypes with the lowest GT50 **(B)** are denoted by letter “a” and genotypes with different letters are significantly different from “a” at 14°C, according to the Wald test at a *p ≤* 0.05. The genotypes with significantly higher and lower root length **(C)** at 20°C are indicated by different letters, according to Wald test at a *p ≤* 0.05. Asterisks indicate significant differences between 14°C and 17°C temperature treatments for each genotype, according to the Wald test at a *p ≤* 0.05. Treatment effects were tested using a REML **(A–C)** differences were considered significant according to the Wald test (p ≤ 0.05).

There was a significant interaction effect of temperature and genotype (*p* < 0.001, Wald test) on germination percentage (**Figure 2A**). Germination was lower (60%) for all genotypes at 14°C compared to 20°C (85%). At 14°C, Bari Mung-4, Satin II and Bari Mung-2 had the highest germination percentage, which was significantly higher than VI064685, VI064686, VI064692, VI064686, VI064692, Green Diamond, Bari Mung-6, Bari Mung-4 and Yezin-9. At 17°C, Bari Mung-3, Bari Mung-4, and Bari Mung-5 had the highest germination with significantly higher germination than VI064685, Emerald, VI064689, Bari Mung-6 and Yezin-1. Jade-AU (check), Bari Mung-2, Satin II and Bari Mung-4 exhibited more than 81% germination across 14, 17 and 20°C. For the reference genotype Jade-AU (check), germination did not differ significantly between 14, 17 and 20°C. In contrast, germination in Celera II-AU (check) significantly increased with increasing temperature (14, 17 and 20°C).

Delta and Emerald exhibited a reduction in germination at 20°C, with germination percentages below 61% and recorded significantly lower germination percentage compared to Putland, Bari Mung-3, Jade-AU, Bari Mung-2, Satin II, and Bari Mung-4. Genotypes Putland, Bari Mung-3, Jade-AU, Bari Mung-2, Satin II, Bari Mung-4 and Bina Mung-8 had no significant differences in germination between 14, 17 and 20°C, with average germination (%) more than 80%.

#### 3.1.2 Percentage of seed germination after sowing

Seed weight (mg) of the seeds selected for germination significantly affected onset of germination at 3 and 5 DAS, becoming insignificant from 7 DAS across the temperatures and genotypes. The number of days post-sowing required for genotypes to germinate was significantly influenced by the interaction of temperature and genotype (*p<*0.001, Wald test), except for 3 DAS (**Table 2**). This interaction led to considerable variation in how genotypes responded to the different temperature treatments. The percentage of seed germination was higher at higher temperatures (**Table 3**). It took at least 5, 7, and 14 DAS to achieve more than 50% change of germination at 20, 17 and 14°C respectively. The effect of temperature was significant on the germination of mungbean seeds at all DAS.

**Table 2:** Wald test *p*-values indicate differences between temperature, genotype and seed weight for germination at different days after sowing (DAS) in a controlled growth environment (Experiment 1).

Source of variation	DAS						
	3	5	7	10	14	17	21
Seed weight (mg)	<0.001	0.025	0.189	0.346	0.582	0.628	0.737
Temperature (°C)	<0.001	<0.001	<0.001	<0.001	0.004	0.006	0.006
Genotype	<0.001	<0.001	<0.001	<0.001	<0.001	<0.001	<0.001
Temperature × Genotype	1.00	<0.001	<0.001	<0.001	<0.001	<0.001	<0.001

**Table 3:** Percentage of seed germination at 14, 17 and 20°C at different days after sowing (DAS) in a controlled growth environment (Experiment 1).

Temperature (°C)	Days after sowing (DAS)						
	3	5	7	10	14	17	21
14	0	2 ± 7.2 c	12 ± 36.4 c	31 ± 52.1 c	51 ± 7.5 b	55 ± 7.2 b	61 ± 6.6 b
17	0	30 ± 5.0 b	53 ± 5 b	68 ± 6.7 b	76 ± 5.6 a	83 ± 19.4 a	85 ± 17.7 a
20	29 ± 4.1	66 ± 5.3 a	86 ± 21.5 a	91 ± 15 a	92 ± 13.6 a	93 ± 12.6 a	94 ± 11.6 a

Mean and standard error values are reported for each temperature treatment for each DAS. Means within a column (DAS) followed by same letters are not significantly different based on LSD test (p<0.05). Both mean and standard error values are back transformed values derived from a generalised linear mixed model (*n* = 4 biological replicates per treatment, with 10 seeds per genotype per replicate, and the entire experiment was conducted in five independent runs).

#### 3.1.3 Effect of low temperature on time to germination

There was a significant interaction between temperature and genotype (*p<*0.001, Wald test) on germination rate (GT_50_) (**Figure 2B**). This resulted in considerable variability among genotypes, ranging from 2 to 16 days for 50% of final germination in response to temperature treatment (**Figure 2B**). The genotypes Bari Mung-3, Bari Mung-4, Bina Mung-6, Bina Mung-7, Bina Mung-8, Yezin-1 and Yezin-11 had lower GT_50_ and the mean GT_50_ was not significantly different among the three temperature treatments. These genotypes were faster to germinate at all test temperatures. In contrast, Emerald and VI064685 were significantly slower to germinate at 14°C, as indicated by their higher GT_50_ values.

#### 3.1.4 Germination percentage and rate

**Figure 3:**
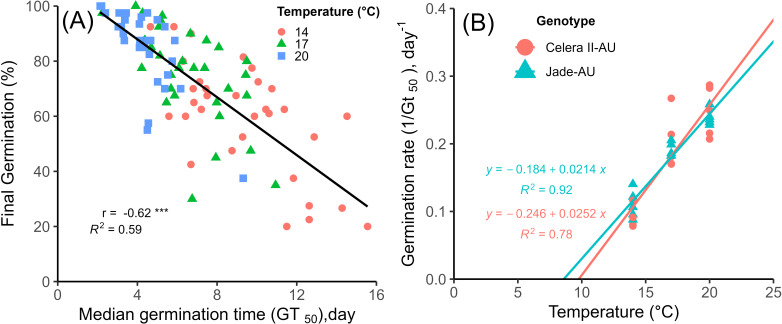
**(A)** Relationship between the final germination (%) and the time to median germination (GT_50_) of mungbean genotypes under controlled growth cabinet at 14, 17 and 20°C. The solid line shows the fitted regression line. Asterisks (***) denote a significant correlation at *p* < 0.001 (Experiment 1). **(B)** Mungbean seed germination rates of Jade-AU and Celera II-AU genotypes at 14, 17, and 20°C (Experiment 1). The solid lines show the fitted regression line. The extrapolation of linear regression back to the temperature axis (x-intercept) gave estimates of base temperature (T_b_) of 8.6 and 9.8°C for Jade-AU and Celera II-AU, respectively. The average temperatures observed during Experiment 1 were 14 ± 0.2, 17 ± 0.1 and 20 ± 0.1°C for the constant temperature treatments of 14, 17 and 20°C, respectively.

A simple correlation analysis between final germination percentage and median germination days (GT_50_) at 14, 17 and 20°C showed that there was an inverse relationship for the tested genotypes (*r*= −0.62**, R^2^ = 0.59) (**Figure 3A**), i.e., the final germination was reduced with increased median days to germination. There were 3, 10 and 20 genotypes with more than 85% final germination within 8 days for 14, 17 and 20°C respectively. The temperature increases from 14 to 20°C increased the germination percentage across genotypes. There was a significant correlation between temperature and germination (%) (*r* = 0.51***), and a negative correlation between temperature and rate of germination (GT_50_) (*r* = −0.69***) at 14, 17 and 20°C. The median time to germinate mungbean seeds was 4.3 days at 20°C, 6.5 days at 17°C and 9.4 days for 14°C.

#### 3.1.5 Base temperature

From the estimates of median germination time (GT_50_), a regression plot of reciprocal GT_50_ (germination rate) against temperature was plotted for a linear relationship for temperature (**Figure 3B**) for reference genotypes Celera II-AU and Jade-AU. The extrapolation of linear regression back to the temperature axis indicated that the base temperatures were 8.6 and 9.8°C for Jade-AU and Celera II-AU respectively.

#### 3.1.6 Effect of low temperature on root length

There was a significant interactive effect of temperature and genotype (*p<*0.001, Wald test) on the root length (mm) (**Figure 2C**). Additionally, there was considerable variability among genotypes, with average root length responses to temperature treatment ranging from 3 to 167 mm (**Figure 2C**). The genotypes Bari Mung-3, Bari Mung-6, and Bina Mung-8 exhibited significantly longer root lengths compared to the reference genotypes Jade-AU and Celera II-AU at 20°C. At 17°C, the root length of genotype Bari Mung-3 was also significantly greater than that of the reference genotypes. However, at 14°C, there was no significant difference in root length compared to the reference genotypes among all the tested genotypes.

### 3.2 Experiment 2: soil-based seed germination and emergence across constant temperatures and soil water status

#### 3.2.1 Constant temperature and emergence

**Figure 4:**
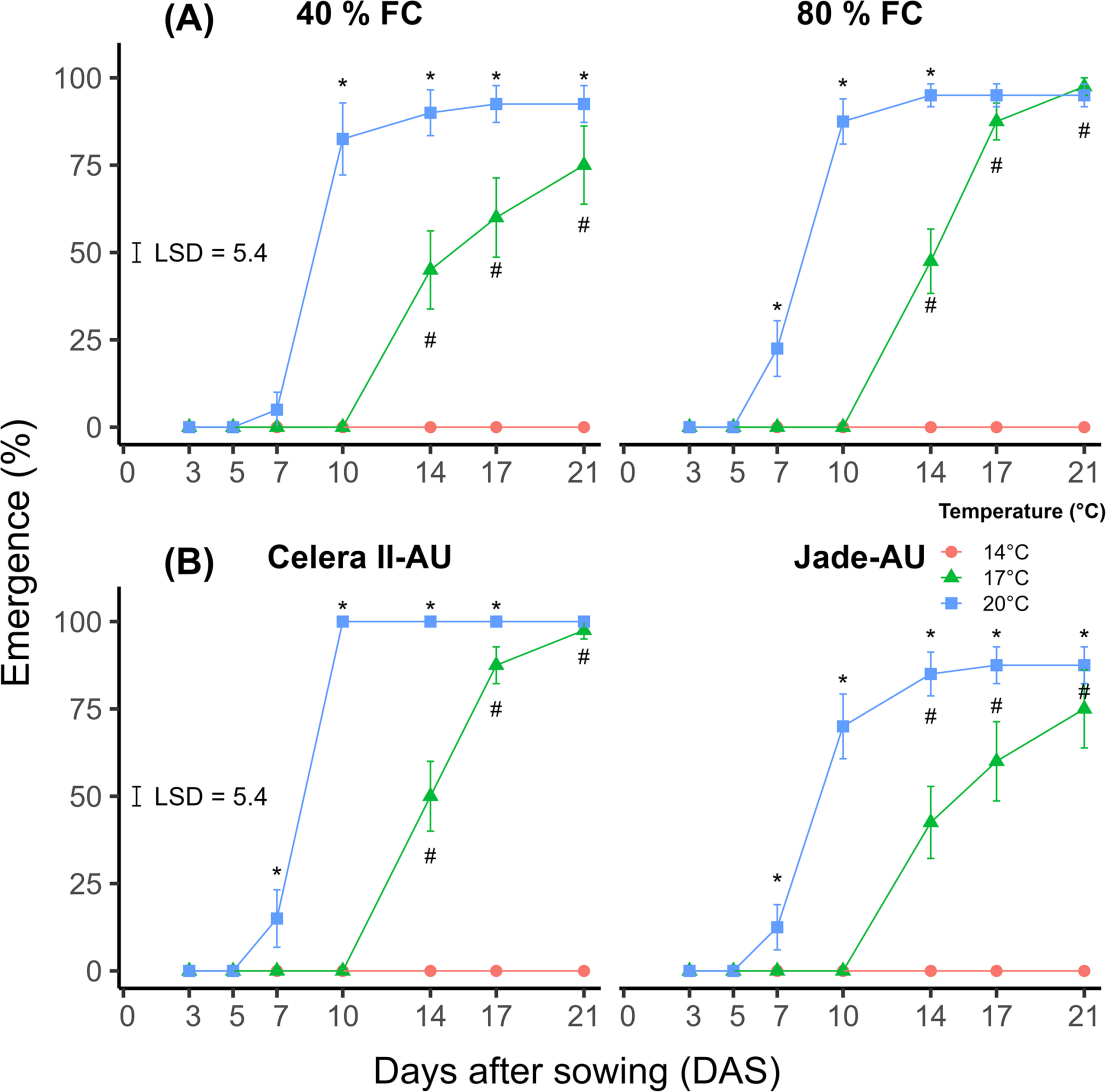
**(A)** The emergence percentage (%) was observed at two soil water treatment levels, 40 and 80% field capacity (FC), and three constant temperatures (14, 17 and 20°C) over a period of 21 DAS (Experiment 2). **(B)** The emergence percentage (%) of Celera II-AU and Jade-AU was recorded at three constant temperatures (14, 17 and 20°C) over a period of 21 DAS (Experiment 2). The observed temperatures (mean ± sd) of constant temperature treatments for 14, 17 and 20°C were 14 ± 0.2, 17 ± 0.3 and 20 ± 0.2°C, respectively, during the Experiment 2. Mean and standard error values of four independent biological replicates (*n* = 4, pool of five plants) from Experiment 2 are presented. The black error bar within the graph signifies the least significant difference (LSD) at a significance level of *p ≤* 0.05 for comparison of **(A)** the temperature × soil water status × DAS interaction (LSD_0.05_ = 5.4) and **(B)** temperature × variety × DAS interaction (LSD_0.05_ = 5.4). Asterisks indicate the significant differences between 20°C and 17°C at each DAS according to LSD test (*p ≤* 0.05). Hashtags indicate the significant differences between 17°C and 14°C at each DAS according to LSD test (*p ≤* 0.05).

The percentage emergence (%) was significantly affected by the temperature × water treatment × DAS interaction (*p<*0.05, LSD_0.05_ = 5.4) (**Figure 4A**). Over the 21-day period, mungbean emergence (%) at 40% field capacity (FC) soil water content was significantly higher at 20°C after 10 DAS compared to 14 and 17°C (**Figure 4A**). However, at 80% FC soil water content the emergence percentage at 20°C was significantly higher at 7 DAS compared to 14 and 17°C. Additionally, the final emergence was not significantly different between 17 and 20°C at 21 DAS with 80% FC water treatment. In contrast, at 40% FC water treatment there was a significant difference between 17 and 20°C for the final emergence. At 40 and 80% FC soil water, the highest average emergence for the 20°C treatment occurred at 17 and 14 DAS. In contrast, it took 21 days to reach peak emergence at 17°C for both soil water treatments.

The percentage emergence was significantly affected by a three-way interaction of temperature, genotype and DAS (*p<*0.05, LSD_0.05_ = 5.4) (**Figure 4B**). Over the period of 21 days, the emergence (%) of Celera II-AU and Jade-AU was significantly higher at 20°C after 7 DAS compared to other temperatures, 14 and 17°C (**Figure 4B**). However, for Celera II-AU at 21 DAS, the difference between 20 and 17°C was not significant. The greatest emergence for Celera II-AU was reached at 10 DAS at 20°C, while for Jade-AU it took 17 days for the highest emergence (%) across the soil water treatments. For both genotypes at 17°C, it took 21 DAS to reach the maximum emergence (%) across the soil water treatments.

At 14°C constant temperature, there was no emergence observed at 21 DAS (**Figures 4A, B**), even though seed germination was observed at 96% at 14°C after washing the soil.

#### 3.2.2 Shoot and root length

**Figure 5:**
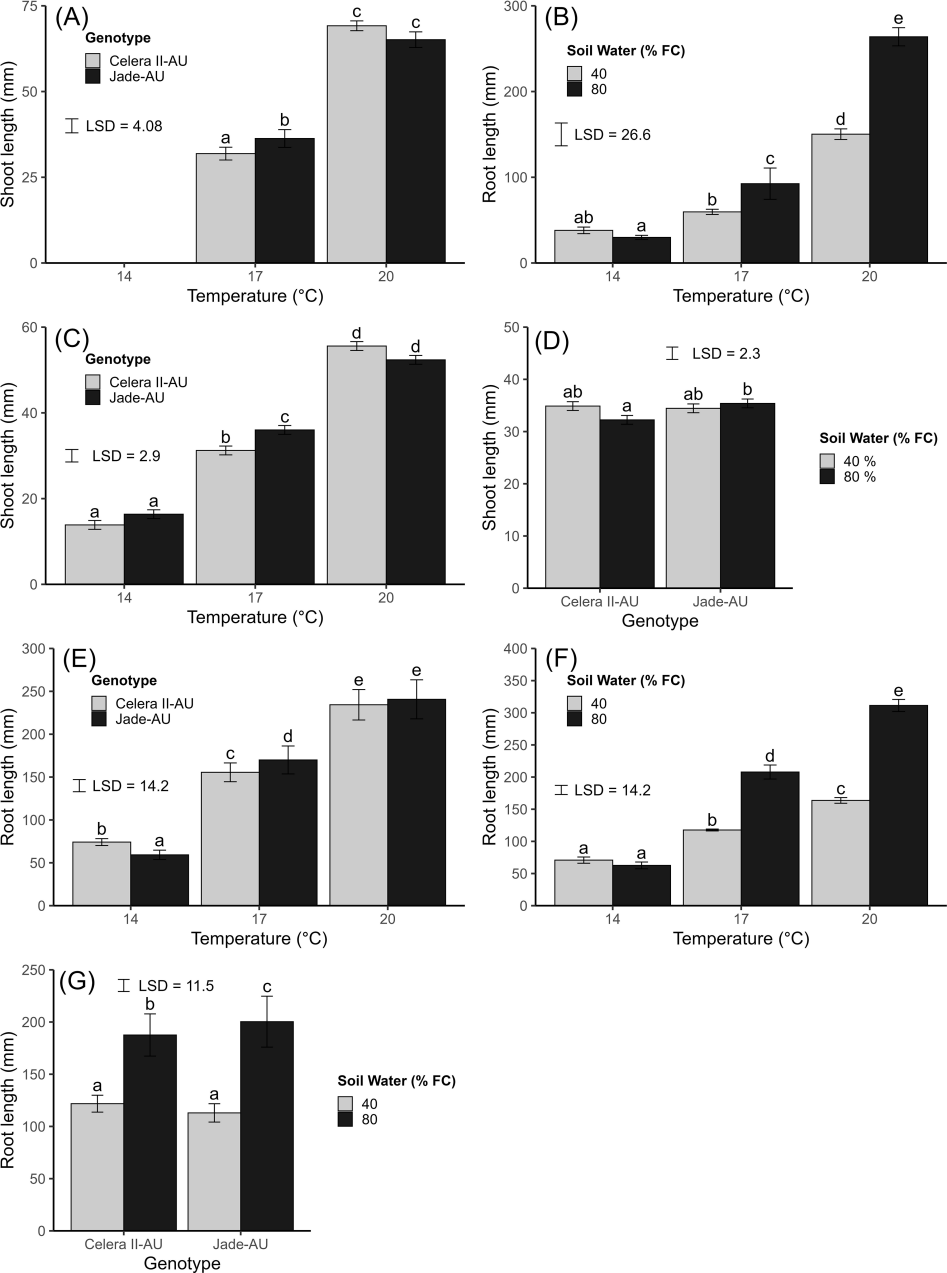
The effect of temperature and soil water status percent of field capacity (% FC) on mungbean genotypes (Celera II-AU and Jade-AU) under controlled conditions where, **(A, B)** = Constant temperature and **(C–G)** = Diurnal temperature. Mean and standard error values of four replicates (*n* = 4, pool of five plants) from Experiment 2 **(A, B)** and eight replicates (*n* = 8, pool of five plants) pooled from two independent experimental repetitions from Experiment 3 **(C–G)** are presented. During the Experiment 2, the observed temperatures (mean ± sd) of constant temperature treatments for 14, 17 and 20°C were 14 ± 0.2, 17 ± 0.3 and 20 ± 0.2°C respectively. While during Experiment 3, The observed mean temperatures (mean ± sd) for diurnal temperatures were 14 ± 3.6, 17 ± 3.6 and 20 ± 3.6°C for the temperature treatments of 14, 17 and 20°C respectively. The black error bar in the graph represents the least significant difference (LSD) at *p ≤* 0.05 for comparisons of the interaction between the factors (temperature, genotype and soil water status) at each figure. Bars followed by different letters differ significantly according to Fisher’s least significant difference (LSD) test at a *p ≤* 0.05. No significant repetition effect was observed in Experiment 3 (F = 0.10, *p* > 0.05).

The shoot length of mungbean was significantly affected by the temperature × genotype interaction (*p=*0.02, LSD_0.05_ = 4.08) (**Figure 5A**). The shoot length of mungbean genotypes was not significantly different at 20°C, but the genotypes were significantly different at 17°C. Jade-AU had a significantly greater shoot length than Celera II-AU at 17°C, whereas at 20°C, Celera II-AU had a greater shoot length than Jade-AU. At 14°C, there was no emergence, and no shoots were observed. There was a significant effect of water treatment (*p* = 0.02, LSD_0.05_ = 2.36) on shoot length (data not presented), with a notably greater shoot length observed for the 80% FC water treatment (35 mm) compared to the 40% FC treatment (32 mm).

The root length was significantly affected by the temperature and water treatment interaction (*p<*0.001, LSD_0.05_ = 26.6) (**Figure 5B**). Root length at 80% FC water treatment was significantly greater at 17 and 20°C compared to 40% FC water treatment, but the root length was not significantly different at 40% FC water treatment.

### 3.3 Experiment 3: soil-based seed germination and emergence across diurnal temperatures and soil water status

#### 3.3.1 Diurnal temperature and emergence

**Figure 6:**
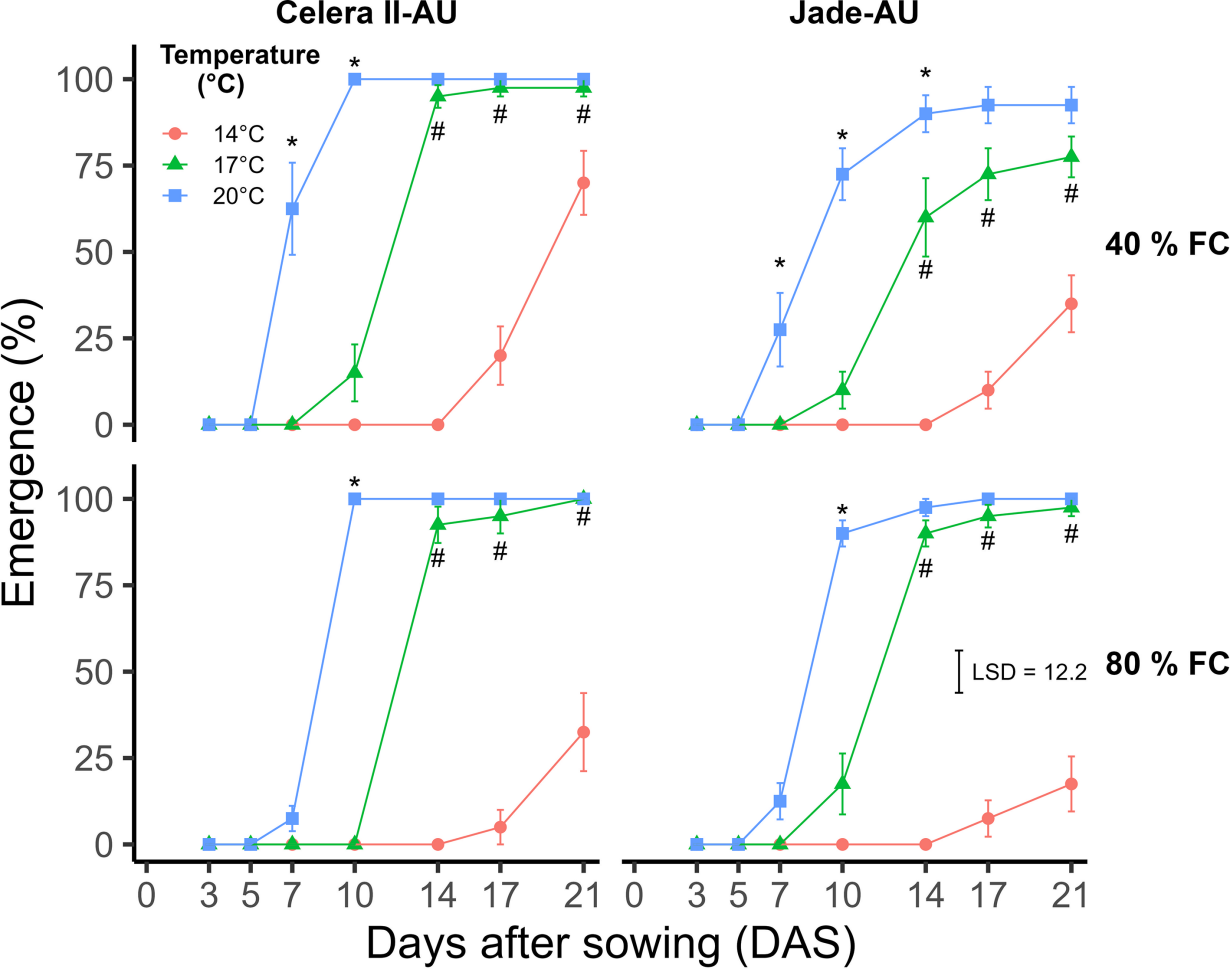
Emergence of mungbean genotypes (Celera II-AU and Jade-AU) grown at two soil water content (40 and 80% of field capacity) and three temperatures over a period of 21 days (Experiment 3). During the experiment, the observed mean temperatures (mean ± sd) for diurnal temperatures were 14 ± 3.6, 17 ± 3.6 and 20 ± 3.6°C for the temperature treatments of 14, 17 and 20°C respectively. Mean and standard error values of eight replicates (*n* = 8, pool of five plants) pooled from two independent experimental repetitions from Experiment 3 are presented. The black error bar within the graph signifies the least significant difference (LSD) at a significance level of *p ≤* 0.05 for comparison of the temperature × genotype × soil water status × DAS interaction (LSD_0.05_ = 12.2). No significant repetition effect was observed (F = 0.10, *p* > 0.05). Asterisks indicate the significant differences between 20°C and 17°C at each DAS according to LSD test (*p ≤* 0.05). Hashtags indicate the significant differences between 17°C and 14°C at each DAS according to LSD test (*p ≤* 0.05).

The emergence of mungbean was significantly affected by the four-way interaction (*p<*0.05, LSD_0.05_ = 12.2) between temperature, water treatment, genotype, and DAS (**Figure 6**). At 7 DAS, the emergence of Celera II-AU and Jade-AU was significantly higher at 20°C compared to 17 and 14°C for the 40% FC water treatment. However, for 80% FC water content the difference between 20°C and the 17 and 14°C treatments became significant at 10 DAS for both varieties. On 14 DAS, the emergence between 20 and 17°C was insignificant across the varieties and water treatment, except for Jade-AU at 40%. Additionally, the difference between 17 and 14°C was significant at 14 DAS. Jade-AU exhibited significantly higher emergence at 20°C compared to 17 and 14°C after 7 DAS, regardless of soil water treatment (**Figure 6**). Celera II-AU demonstrated the highest emergence, plateauing at 10 DAS for 20°C. At 17 and 14°C, the emergence peaked at 21 DAS for Celera II-AU. In contrast, Jade-AU required 21 DAS to achieve maximum emergence across the temperature and soil water treatments. The emergence of Celera II-AU exceeded 92% in 14 DAS at 17°C and surpassed 97% in 21 DAS, regardless of the soil water treatment. In contrast, at 14°C, the highest emergence was less than 50% across the different varieties, temperatures, and soil water content treatments, except for Celera II-AU which attained 70% emergence at 40% FC soil water content (70%). The final emergence was highest at 20°C across the treatments; however, it was not significantly different from that at 17°C.

#### 3.3.2 Shoot and root length

The shoot length of mungbean was significantly affected by the genotype × temperature interaction (*p<*0.02, LSD_0.05_ = 2.9) (**Figure 5C**). The shoot lengths of mungbean genotypes were not significantly different at 14 and 20°C, but they were significantly different at 17°C. Jade-AU had significantly greater shoot lengths than Celera II-AU at 17°C.

The shoot length was significantly affected by the genotype and water status interaction (*p=*0.038, LSD_0.05_ = 2.3) (**Figure 5D**). At 80% FC water status, Jade-AU had significantly greater shoot length than Celera II-AU, while at 40% water status shoot length was not significantly different between varieties or genotypes.

For root length, there was a significant interaction between temperature and genotype (*p=*0.046, LSD_0.05_ = 14.2) (**Figure 5E**). The root length was not significantly different at 20°C between Jade-AU and Celera II-AU. In contrast, at 14°C root length was significantly greater for Celera II-AU compared to Jade-AU, whereas at 17°C Jade-AU had greater root length than Celera II-AU.

The root length was also significantly affected by the temperature × water status interaction (*p<*0.001, LSD_0.05_ = 14.2) (**Figure 5F**). Root length at 80% FC water status was significantly greater at 17 and 20°C compared to 40% FC water status but was not significantly different at 40% FC water status.

The root length of genotypes was significantly affected by water status (*p<*0.001, LSD_0.05_ = 11.5) (**Figure 5G**). At 80% water status, Jade-AU’s root length was significantly greater than Celera II-AU’s. However, at 40% water status root length was not significantly different between genotypes.

## 4 Discussion

Soil temperature during the sowing of mungbean has a significant effect on germination, emergence, vigour and ultimately crop establishment (Simon et al., 1976; Fyfield and Gregory, 1989; Amin et al., 2023). This study demonstrates that lower temperatures significantly reduced germination, emergence and seedling vigour (root and shoot) of mungbean genotypes under controlled conditions.

Previous studies have shown that increasing temperature from 25 to 35°C increases the germination rate of mungbean (Amin et al., 2023) and that germination is optimised at temperatures of 30–40°C (Fyfield and Gregory, 1989). There have been limited studies examining the effect of temperatures below 20°C, although the findings of Simon et al. (1976) indicated slower germination at temperatures below 20°C. Information on mungbean germination at lower temperatures is important when considering the suitability of cultivating mungbean in more temperate climates. In the present study, the 32 mungbean genotypes – which represent the core collection of AGG sourced from Bangladesh, Myanmar, Australia and India – when tested at 14, 17 and 20°C showed considerable variability in germination. Germination rates were generally higher at 20°C and declined significantly at lower temperatures. There was genetic variation, with some genotypes (Bari Mung-2, Satin II and Bari Mung-4) having improved germination at cooler temperatures, indicating that these genotypes possess a degree of cold tolerance compared to the other genotype tested, including Australian commercial genotypes. The genotypes Bari Mung-2 and Bari Mung-4, developed in Bangladesh (Afzal et al., 2001; BSMRAU, 2024), may be recommended in areas with low soil temperatures during sowing time after testing in field trials due to their potential adaptation to colder regions compared with other genotypes. Additionally, these genotypes have potential to be used in breeding programs to improve cold tolerance of mungbean during germination.

The base temperatures for germination were 8.6 and 9.8°C for Jade-AU and Celera II-AU respectively. The base temperature observed in this study is lower than the 10.1°C estimated by Fyfield and Gregory (1989) for the germination of mungbean when water is not a limiting factor. In studies of other crops, such as soybean (Szczerba et al., 2021), germination at low temperature (i.e., below 10°C) was severely decreased.

Mungbean genotypes germinating in cold conditions do not have sufficient vigour to establish well. While some genotypes show higher germination rates and lower base temperatures, their final root lengths at 17 and 14°C were significantly reduced. This indicates that seeds can germinate at low temperatures, but further root growth is inhibited. Root length and seedling emergence will be crucial for the establishment and successful cultivation of mungbean in southern Australia as an opportunistic crop. Furthermore, due to rising temperatures after sowing, genotypes that can germinate at lower temperatures may establish earlier than those needing higher temperatures, as they may have already accumulated the necessary thermal units for the next growth stage. The radicle requires specific cumulative temperatures not only for germination but for each growth stage (Shaban, 2013; Haj Sghaier et al., 2022). Therefore, the temperature response of mungbean genotypes may differ during germination due to its complexity involving many phases and reactions (Shaban, 2013).

In pot experiments, seedling emergence was faster and higher for Celera II-AU than Jade-AU across both diurnal and constant temperature conditions. Both genotypes, Celera II-AU and Jade-AU, emerged at diurnal temperatures of 20°C, 17°C, and 14°C and constant temperatures of 20 and 17°C, but failed to emerge at a constant temperature of 14°C, despite germination occurring. Seedling emergence above 20°C was reported by Fyfield and Gregory (1989), however, the effect below 20°C were not reported well before. The lower emergence rate below 20°C and failed emergence at 14°C is likely due to prolonged cold stress damaging the mitochondrial structure, which reduces kinetic energy flow and disrupts enzyme activity, leading to a decreased respiration rate (Ikkonen et al., 2020). This causes permanent damage, although the plant may recover from short-term cold stress.

Soil temperature is a vital factor affecting root growth and development. Root elongation was greatest at 20°C and declined at lower temperatures (17 and 14°C). The reduction in temperature has a negative impact on root growth, as indicated by lower measurements at these temperatures. Although seeds germinated and the radicle grew slightly at lower temperatures, overall growth stalled (Experiment 1 root data). While temperatures above the optimal level may accelerate germination, this does not necessarily result in sustained root growth over time. These results align with Khaeim et al. (2022), who demonstrated that temperature plays a crucial role in initiating germination in wheat, as seeds absorb heat units from the available thermal energy. Once they reach the threshold for initiating metabolic activity, germination begins at a rate dependent on the temperature at which they are growing. The rate of germination and subsequent growth is temperature-dependent, and different cumulative temperatures at each stage influence the radicle’s development (Haj Sghaier et al., 2022). The sensitivity of mungbean root growth to lower temperatures results from the cumulative temperature requirement for each growth stage, as evident in the higher growth rate at diurnal temperatures compared to constant temperatures (Experiments 1 and 2). The ‘cumulative temperature requirement’ concept refers to the total thermal units needed for a seed to complete its germination process. The complexity of the germination process results in temperature responses varying throughout the germination period (Shaban, 2013). Therefore, the faster growth rate of mungbean genotypes is related to the higher maximum temperatures observed at diurnal temperature treatment, even though the average temperature was the same as in the constant temperature treatment during the experiment.

Water absorption by the seed is a critical step for germination (Luna and Chamorro, 2016). It is essential for enzymatic activity followed by breakdown, translocation, and utilisation of stored protein, carbohydrate and fat (Woodstock, 1988; Ali and Elozeiri, 2017). Increasing soil water content from 40 to 80% FC increased emergence of mungbean across all temperature treatments, with the exception of those grown at diurnal temperature compared to 14°C constant temperature (**Figures 4** and **6**). The emergence percentage increased at 17 and 20°C temperatures (at both constant and diurnal). The higher emergence rate at higher temperatures at 80% FC water could be due to the synergistic effect of favourable temperature and water availability (Ramírez-Tobías et al., 2014; Luna and Chamorro, 2016). In contrast, at 14°C diurnal temperature, the emergence rate at 80% FC compared with 40% FC might be affected by the depleted oxygen at higher water availability. Oxygen availability is crucial for seed germination (Pan et al., 2021). Seeds in dormancy require water, oxygen, and an appropriate temperature to germinate. At 14°C diurnal temperature with 40% FC, due to the lower evapotranspiration rate, the oxygen level could have become more favourable for germination and emergence, which demands further investigation for mungbean genotypes under varying temperate conditions.

In southern Australian cropping, early spring soil temperatures significantly influence mungbean establishment, with cooler conditions impeding germination and resulting in slower emergence and uneven establishment, leading to reduced yield (Hussain and Khalil, 2004; Tzudir et al., 2014; Silwal et al., 2025). To mitigate these effects, mungbean should be sown when soil temperatures exceed 16°C, which is essential for uniform germination and faster crop establishment (Christy et al., 2022). In the context of southern Australian farming systems, in northern Victoria the soil temperature exceeds 16°C in October, while in southern Victoria it reaches 16°C in late November (Delahunty et al., 2020). Based on our study, temperatures below 17°C are likely to delay plant establishment, similar to the field trials in which lower soil temperatures led to longer emergence times and consequently lower plant density (Silwal et al., 2025). Therefore, the favourable time for seed sowing for Victorian conditions is at the borderline between spring and summer. The variation in response to germination of mungbean genotypes to soil temperature at spring sowing times is important, and the identification of genotypes tolerant to lower temperatures during germination and establishment is crucial for the expansion of mungbean cultivation into temperate regions, such as southern Australia, particularly Victoria.

## 5 Conclusion

Temperature significantly influences the germination of mungbean genotypes. The germination rate was highest at 20°C for the majority of genotypes; however, considerable variability was observed among the genotypes at lower temperatures of 17 and 14°C. Notably, the genotypes Bari Mung-4 and Bari Mung-3 exhibited higher germination percentages and faster germination rates across the tested temperatures, showing the greatest cold tolerance along with the genotypes Putland, Satin II, and Bina Mung-8, which showed no significant differences among the treatments at 14, 17, and 20°C, with average germination percentages of 82, 94, and 80 respectively. Mungbean genotypes Celera II-AU and Jade-AU demonstrated increased germination percentage at higher soil water levels and elevated temperatures when subjected to variable soil water (40 and 80% FC). There exists an opportunity for improvement in varietal adaptation to lower soil temperatures during spring sowing conditions in temperate regions. This study increases our understanding of mungbean response to lower soil temperatures and variable water availability. To expand mungbean cultivation to cooler regions such as southern Australia, it will be imperative to study the mechanisms of cold tolerance, particularly in relation to capacity to germinate and establish in cooler soils.

## Data Availability

The original contributions presented in the study are included in the article/**Supplementary Material**. Further inquiries can be directed to the corresponding author.
