# Supplementary material for: Critical temperature requirement for the germination and establishment of mungbean (Vigna radiata L.) in temperate environments
Source: Front Plant Sci. 2026 Feb 6;17:1693408. doi: 10.3389/fpls.2026.1693408 (PMC12921486; doi:10.3389/fpls.2026.1693408)
Supplement: Supplementary file 1 [file Supplementaryfile1.docx]

Supplementary Material

## Supplementary Figures


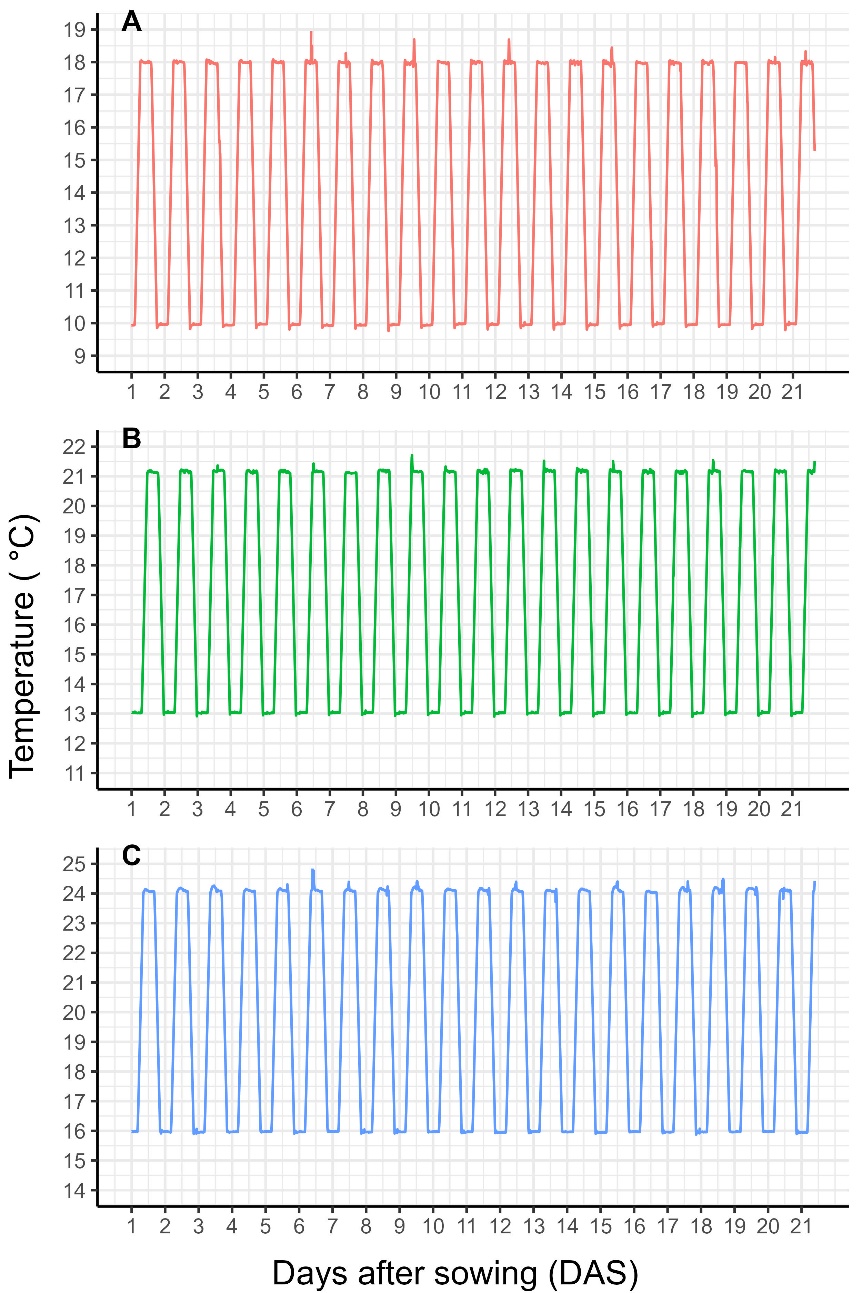


**Supplementary Figure 1.** Temperature recorded inside controlled growth cabinet during the experiment across 21-day period for diurnal temperature regimes: (A) 10 to 18°C, (B) 13 to 21°C, and (C) 16 to 24°C.

**
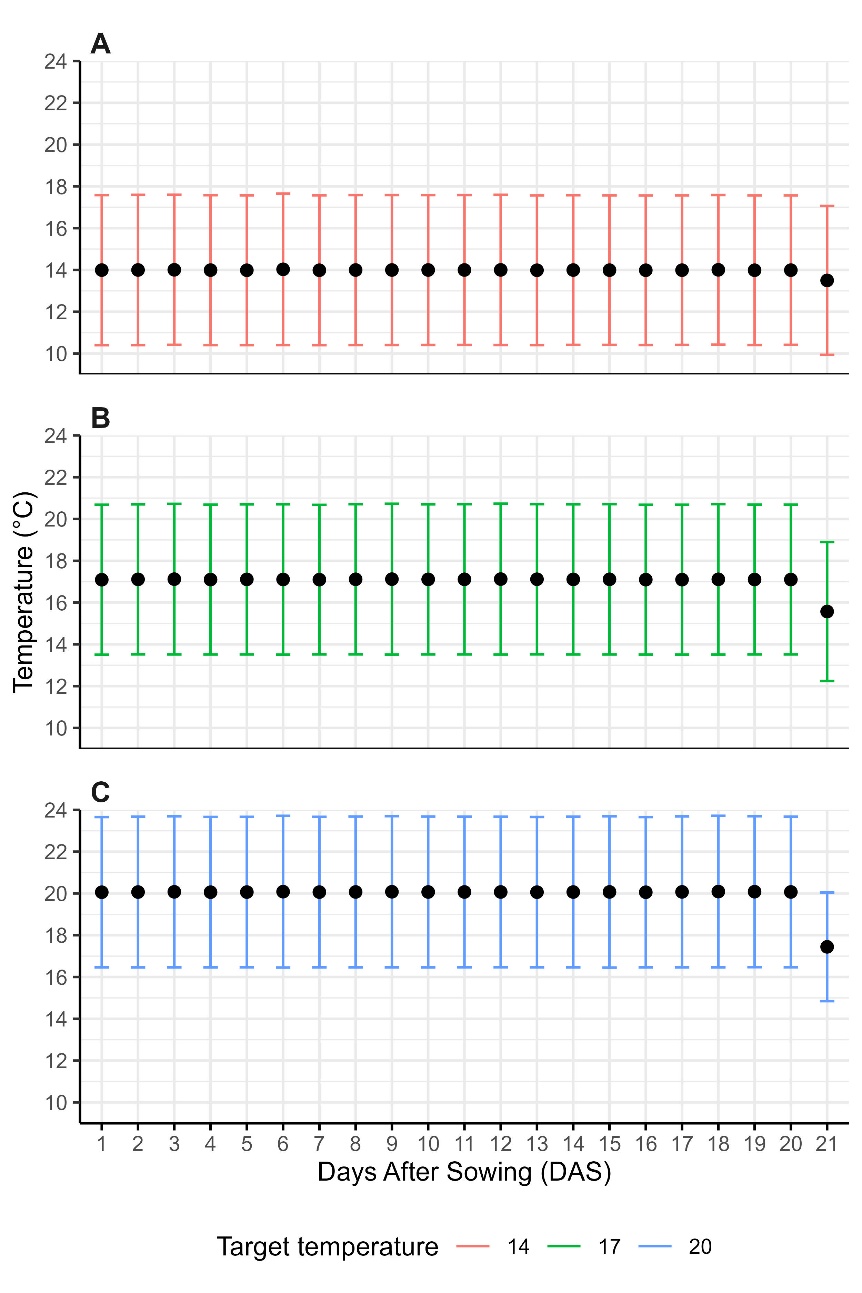
**

**Supplementary Figure 2.** Average temperature recorded inside controlled growth cabinet during the experiment across 21-day period for diurnal temperature regimes: (A) 10 to 18°C, (B) 13 to 21°C, and (C) 16 to 24°C. The diurnal temperature regimes A, B and C were targeted for the mean temperatures of 14, 17 and 20°C. Error bars represent the standard deviation.
